# Coding Locations Relative to One or Many Landmarks in Childhood

**DOI:** 10.1371/journal.pcbi.1007380

**Published:** 2019-10-28

**Authors:** James Negen, Linda Bou Ali, Brittney Chere, Hannah E. Roome, Yeachan Park, Marko Nardini

**Affiliations:** 1 Department of Psychology, Durham University, Durham, United Kingdom; 2 Department of Psychiatry, American University of Beirut Medical Center, Beirut, Lebanon; 3 Department of Psychological Sciences, Birkbeck, London, United Kingdom; 4 Center for Learning and Memory, University of Texas at Austin, Austin, Texas, United States of America; 5 Department of Psychology, University of Amsterdam, Amsterdam, Netherlands; University College London, UNITED KINGDOM

## Abstract

Cognitive development studies how information processing in the brain changes over the course of development. A key part of this question is how information is represented and stored in memory. This study examined allocentric (world-based) spatial memory, an important cognitive tool for planning routes and interacting with the space around us. This is typically theorized to use multiple landmarks all at once whenever it operates. In contrast, here we show that allocentric spatial memory frequently operates over a limited spatial window, much less than the full proximal scene, for children between 3.5 and 8.5 years old. The use of multiple landmarks increases gradually with age. Participants were asked to point to a remembered target location after a change of view in immersive virtual reality. A k-fold cross-validation model-comparison selected a model where young children usually use the target location’s vector to the single nearest landmark and rarely take advantage of the vectors to other nearby landmarks. The comparison models, which attempt to explain the errors as generic forms of noise rather than encoding to a single spatial cue, did not capture the distribution of responses as well. Parameter fits of this new single- versus multi-cue model are also easily interpretable and related to other variables of interest in development (age, executive function). Based on this, we theorize that spatial memory in humans develops through three advancing levels (but not strict stages): most likely to encode locations egocentrically (relative to the self), then allocentrically (relative to the world) but using only one landmark, and finally, most likely to encode locations relative to multiple parts of the scene.

## Author summary

As children get older, they develop better ways to store information in memory. Here we investigate one key aspect of this: how they remember locations in a scene. We asked children from 3 to 9-years-old to remember a target location inside a virtual reality (VR) scene, and then to point to it after they had been ‘teleported’ to a new location within the scene. Young children in particular often made a certain kind of (relatively minor) error. They usually responded near the correct landmark, but had trouble correctly orienting their responses around this landmark, which would require them to also make use of the other landmarks. Our interpretation is that they do not necessarily store the full scene in memory. On most trials, they seem to store only the area immediately around the target. Formally, we made a mathematical model of this algorithm and used cross-validation to show that it holds better than alternative explanations. This provides new depth to our understanding of the different ways that memory changes during childhood. An important avenue for future research is testing the potential of VR tasks of this kind for screening and interventions to improve childhood science and math education.

## Introduction

Spatial cognition is a skill that humans and many other organisms employ almost constantly. Because human development is a particularly long process, we have to understand and make decisions within the space around us for years before reaching full cognitive maturation. Potential applications such as screening and interventions to promote spatial-cognitive development during early childhood, which have been identified by educators as a major unfulfilled need [1], require a strong understanding of the typical structure of developing spatial cognition. To this end, many studies have examined the distinction and interplay between egocentric (self-based) and allocentric (world-based) spatial memory [2–13], finding consistently that allocentric memory is a distinct cognitive process with a higher level of difficulty. Previous developmental studies have asked which kinds of cues allow access to allocentric recall at different points in development (e.g. coincident cues [14], beacons [15], proximal landmarks [16, 17], distal landmarks [15, 16], salient landmarks [18], unstable landmarks [19], language [20, 21], transparent boundaries [22], and geometric relations [14, 23–25]). Another crucial way to subdivide allocentric reasoning is by the richness of the representation, remembering a target location relative to one landmark versus many. Here we present new data and new models to further probe a key question from previous work [21, 26–30]: do children use multiple landmarks to encode a target location allocentrically? How does this tend to change across development?

Previous work has largely concluded that during allocentric recall, young children remember locations as a set of allocentric relations to multiple landmarks, rather than just a single landmark [21, 26, 28–30]. Biology researchers working with non-human species pioneered the expansion paradigm to test this issue [31]. In this paradigm, a target is hidden in the middle of two or four landmarks. After training, the array of landmarks is expanded for a test trial. If the target location was coded and recalled as ‘in the middle’, then test trial searches should still be in the middle of the expanded landmark array. That would necessarily involve the use of multiple landmarks. If the target location was instead encoded and recalled as a vector from one landmark, then test trial searches should retain that vector. For example, suppose the middle of the pre-expansion landmark array was 50cm southwest of the northeast landmark. If that is how the location was recalled, then the test trial searches should still be 50cm southwest of the northeast landmark, even though this is no longer ‘in the middle’. This would involve only using one landmark. Variations on this paradigm have been applied to human children as well, from approximately 2 to 8 years old. Most reports conclude that children encode the locations as ‘in the middle’ [21, 26, 28–30] (while another found a pattern of results that was not particularly consistent with any hypothesized strategy [27]). This fits into a broader theoretical context in which young children’s behaviour indicates they use multiple sources of information in concert to make decisions (e.g. metric and categorical spatial information [32, 33], inferring causes by using multiple trials or presentations [34], and the McGurk effect [35]).

However, the current literature leaves a gap wherein the available results concern themselves with the particular relation ‘in the middle’ and do not test a wider variety of possible spatial relations [21, 26–30]. The ‘in the middle’ relation is interesting because it has special language (at least in English), but the exclusive use of targets in the middle of the landmark array unfortunately leaves open a third interpretation. Regardless of how the child encodes the target, if they recognize that the landmark array has changed after training, they may not know how to proceed and may instead just search in the middle because of a response bias [36, 37]. In other words, it may be a kind of ‘default’ place to search when the child does not know where to search. The present study aims to learn more about this cognitive process by using a new paradigm that allows for the presentation of many different target locations. Our new paradigm provides a new way to diagnose the number of landmarks used in the recall process.

**Fig 1:**
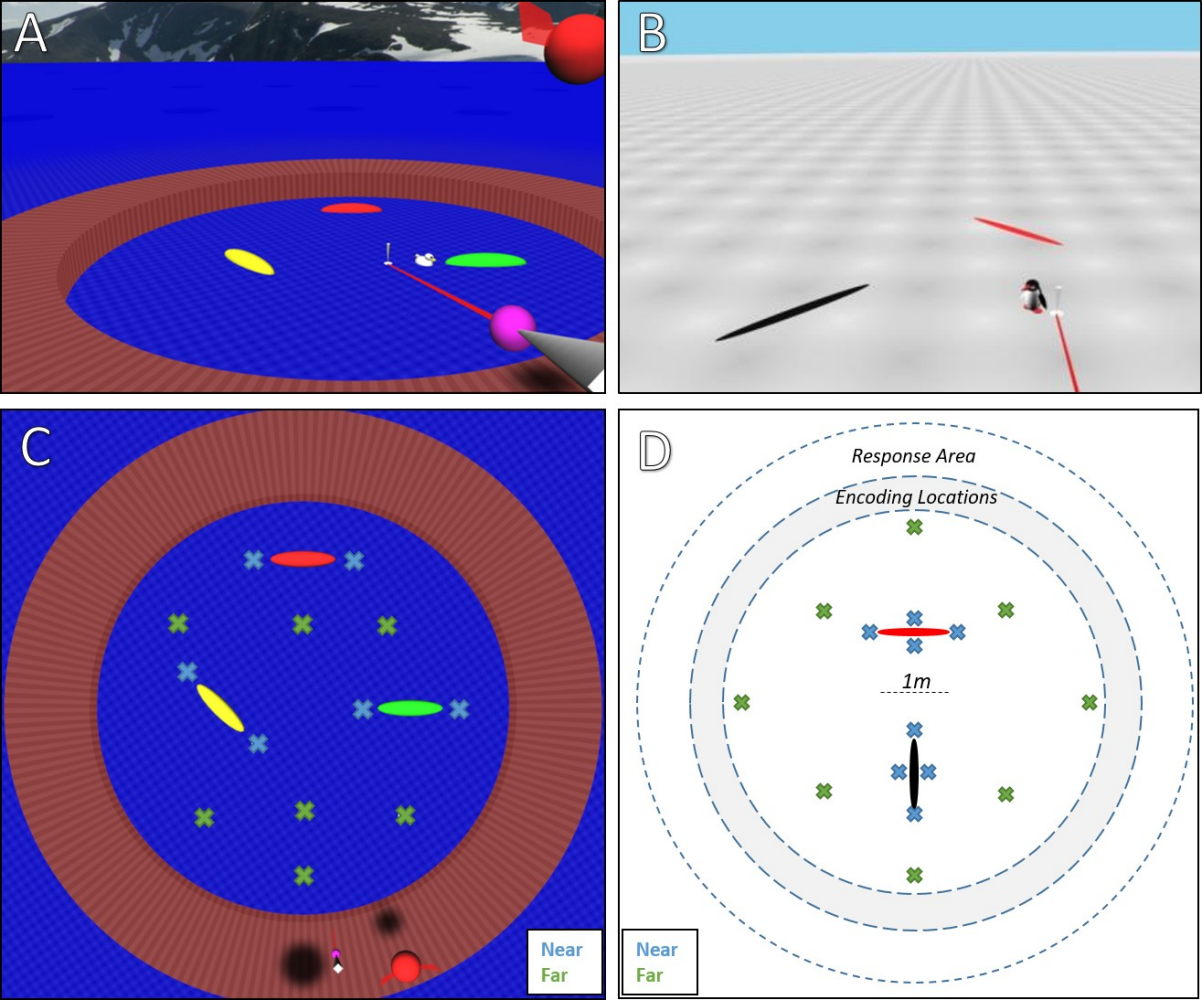
Virtual layouts and target distribution in task. The basic task was to remember where a virtual cartoon animal (duck or penguin) was hiding, watch it disappear, get ‘teleported’ to a new viewpoint, and then point with the ‘magic wand’ to its hiding place. Panels A and B are screenshots of the environments. Panels C and D are overhead layout diagrams of the landmarks and targets. The trials that use the ‘near’ targets are the only ones considered here (in blue) since the ‘far’ trials (in green) were extremely noisy for the youngest age group. We will refer to these as the ‘Jetty’ (panel A and C) and ‘Arctic’ (panel B and D) datasets.

We designed two novel experiments to allow for specific predictions that can disambiguate if one or many landmarks are being used (Fig 1). Crucially, the environment in both experiments had symmetrical oval landmarks. Participants were shown the target location, virtually ‘teleported’ to a new viewpoint (screen fades to black, camera moves, screen fades back to scene), and then asked to point to the target location. This ensured that egocentric reasoning could not contribute to improvements in performance. The use of symmetric landmarks (ovals) meant that a single vector between the target and a nearby landmark would not provide any way to resolve the local symmetry. In the extreme case, when using only a single-cue strategy (one landmark), we would expect an equal number of responses at the correct location and at its mirror across the local landmark. For example, in Fig 1A, how could you remember which end of the green oval the target (duck) is on? Relating the target location to the green oval would not be enough. The two ends of the green oval are local mirrors of each other. If that were all you remembered, you would often respond by the wrong end. Consistently disambiguating the two ends would requires the use of a multi-cue strategy (2 or more landmarks). For example, you could remember that it is on the end of the green oval (cue 1) and also remember how far it is from the boundary (cue 2). The two different environments represent two broad strategies for enabling good performance: (1) only presenting two landmarks, so that there is minimal distraction, and (2) presenting a rich environment with a large boundary [38], so that there are several possible ways to encode multiple spatial relations.

From a certain point of view, the present study is similar to studies where a single uniquely colored wall (cue 1) can disambiguate the rotational symmetry of a rectangular enclosure (cue 2) [39]. However, the theoretical interpretation is not exactly the same. Young children can use a colored landmark in a left/right sense [23], meaning that they do not strictly need the rectangular enclosure to find the correct location (see also [20]). The present study builds on this by creating a new situation where no single landmark can be used to uniquely encode the correct target location. Like any change in methods, this could lead to largely different results. In particular, the present study requires the child to go beyond selecting the single best cue and instead requires coordination between multiple individually-ambiguous cues to consistently find the correct target.

To understand the response patterns, we propose a model that has separate parameters for the rates of remembering where target locations were presented relative to (a) the nearest single proximal landmark and (b) additional proximal landmarks. We will refer to this as the Single- and Multi-Cue Model. Purely doing one or the other would represent a qualitative difference in how a location is remembered, fundamentally capturing just one single highly-localized vector (single-cue) versus representing an interconnected graph of a larger scene (multi-cue). In our model, the parameters can be set so that the single-cue strategy is used frequently. This can capture and predict a pattern of responses that appear correct if only looking at a small area around the target–responses that can be identified as incorrect when looking at the larger scene. To test the hypothesis that this feature is necessary to model and understand developing spatial cognition, we compared the ability of several different models to explain the data from the two experiments.

The Single- and Multi-Cue Model has three critical parameters. The first two are local, single-cue parameters. The chance of remembering the correct landmark is p_1_. The chance of remembering if the target was on the end or side of the nearest landmark is p_2_. A very high p_1_ and p_2_ can be achieved without encoding more than the relations to the nearest single landmark. The last parameter is the multi-cue parameter. The chance of using other scene features to disambiguate the local symmetry (for example, remembering that the duck in Fig 1A is on the inner end of the green landmark, not the end nearer the boundary) is p_3_. Since these are independent parameters, this model can flexibly capture different rates of the different allocentric sub-types. (Note that this does not imply classifying individual children into hard stages; rather, it describes the frequency of different strategies within an age range.) However, this also involves additional parameters and a new theoretical commitment, so we want to be as sure as possible that we cannot understand the data sufficiently with fewer parameters and simpler mechanics.

**Fig 2:**
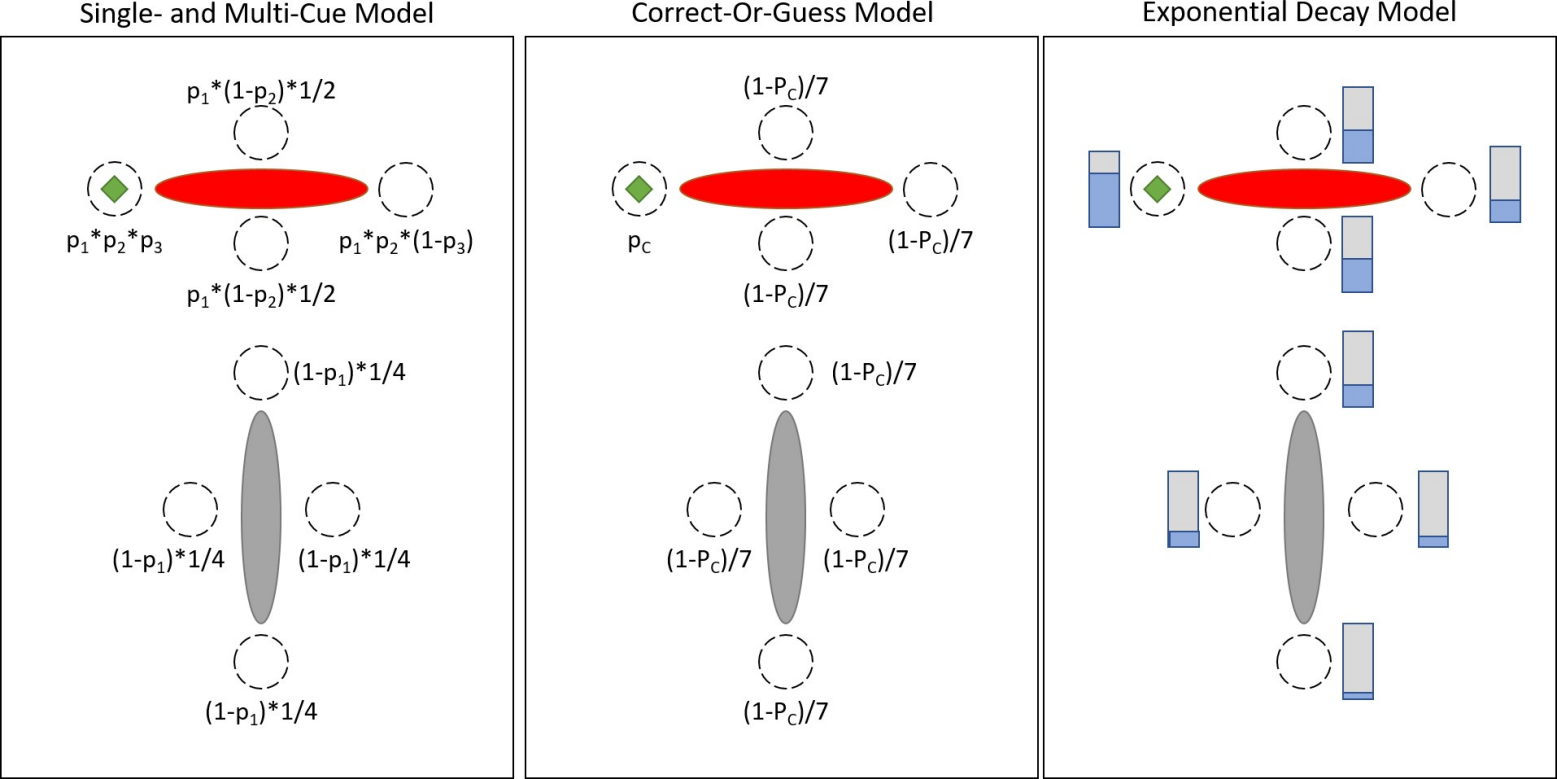
Graphical depiction of how the proposed model (left) and generic-noise alternatives work. The green diamond is the correct target. All three are (bivariate) Gaussian mixture models, with the mixed Gaussians (dotted lines) centered on the 8 potential targets. On the left panel, the Single- and Multi-Cue model uses three binary parameters to select the Gaussian to draw from. The first (p_1_) is how often they respond near the correct landmark, the second (p_2_) is how often they remember if it was on a side versus an end, and the third (p_3_) is how often they use the other landmark to disambiguate the local symmetry. In the middle panel, the correct Gaussian is chosen with probability p_c_ and all other Gaussians share (1-p_c_) equally. This allows for noise but not for a particular weight to errors from local mirroring. In the right panel, the probability of a Gaussian being drawn from is proportional to e^-(kd)^, where *d* is the distance in meters and *k* is a decay rate parameter. This also allows for noise, but bases it on distance rather than mirroring relations. (This is still a Gaussian mixture model, though; it still has 8 local maximums).

To test our proposed Single- and Multi-Cue Model, it is compared against two generic-noise models, Correct-Or-Guess and Exponential Decay, and 315 structured-noise models. The generic-noise models use only one parameter to capture generic noise, in line with conclusions against the use of a single-cue strategy [21, 28–30]. The Correct-Or-Guess model has a single probability that they remember the correct area. If this fails, they guess randomly. This is captured by assigning the correct target a probability of p_c_, then assigning all other targets an equal portion of (1-p_c_). In the Exponential Decay model, response areas become less likely as they get further from the correct target. This is captured by the expression e^-kd^, with the probability of a response decreasing exponentially as the distance from the target (*d*) increases. In other words, these models try to explain putative single-cue errors as not being special in any way, just a random guess or a response area that was relatively near the correct target (Fig 2). The 315 structured-noise models are a computer search of the space of models that use the same number and type of parameters as the Single- and Multi-Cue Model, checking to make sure that there is not a better model at the same level of complexity. These models are then all compared by cross-validation. The central alternative hypothesis is that one of these comparison models will be preferred by cross-validation (highest joint probability of the testing data), suggesting that the single- versus multi-cue distinction is unnecessary, in line with current theory that is based much more on multi-cue strategies.

In addition, the proposed single- and multi-cue model is fit by Bayesian Markov Chain Monte Carlo methods. The posterior parameter distributions are then used to answer several important secondary questions: What are the major sources of error in allocentric spatial memories at different ages? How can we now characterize the progression towards adult-like spatial memory? Were there any specific errors that were more likely to occur for older children? Was memory performance different in these two environments and does this point towards any possible new ideas for further testing? Can we explain individual differences in spatial memory development with more general cognitive measures–especially inhibition, which could play a role in suppressing less-accurate response strategies? The ability to answer each of these questions using the Single- and Multi-Cue Model speaks further to this theory’s usefulness as a way of understanding the development of allocentric spatial memory.

## Results

Participants of all ages gave responses that were well above chance accuracy. In the ‘Arctic’ dataset, the environment was 2.5m in radius and the median error was 61cm for the youngest age group (3.5–4.5 years old). They responded to the correct nearest landmark on 77% (406/528) of trials, p < .001 versus 50% chance guessing, with the 4.5–5.5 year-olds scoring 88% (492/556). For the ‘Jetty’ data, the 4.5–5.5 year-olds responded to the correct nearest landmark on 69% (116/168) of trials, p < .001 versus 33.3% chance guessing, with the next two age groups scoring 67% (140/210) at 5.5–6.5 years and 97% (122/126) at 6.5–8.5 years. The ‘teleporting’ procedure ensured that participant’s performance levels were not possible via purely egocentric encoding. While participants were broadly capable of encoding the target locations allocentrically, even in the youngest age group, a broad range of errors were still evident, motivating a modelling approach.

Cross-validation pointed strongly to the use of the Single- and Multi-Cue Model over all of the alternatives as a way of explaining allocentric encoding in children. Fig 2 displays the ‘Arctic’ dataset to illustrate the proposed model and the two generic-noise alternatives function. Table 1 shows the cross-validation scores; the single- and multi-cue model had the best score for each age group and experiment. All three models were Gaussian mixture models, centered on the eight targets. The cross-validation procedure found maximum likelihood estimates of the parameters using the data from all-but-one target (the training data). It then used these parameters to predict the data from the remaining target (8-fold for the ‘Arctic’ dataset, which had 8 targets, and 6-fold for the ‘Jetty’ dataset, which had 6 targets). This was repeated until each point of data had been predicted once for each model. The score given is the negative sum of the logarithm of the probabilities assigned to the testing data (the part of the data left out of the fitting).

**Table 1:** Table of cross-validation scores.

Experiment and Age	Cross-Validation Score		
	*Single vs Multi*	*Correct or Guess*	*Exponential*
Arctic			
3.5–4.5 Years	**887.64**	928.00	900.68
4.5–5.5 Years	**555.76**	683.28	642.39
Jetty			
4.5–5.5 Years	**329.79**	390.85	339.62
5.5–6.5 Years	**467.17**	522.59	471.78
6.5–85. Years	**-7.80**	28.96	-1.32

*Note*. Lower scores indicate better performance. Figures are the negative sum of the log of the probability assigned to the dataset. For reference, a difference of 4.6 between two scores translates to approximately 100 times better prediction overall. Best scores are in bold.

**Fig 3:**
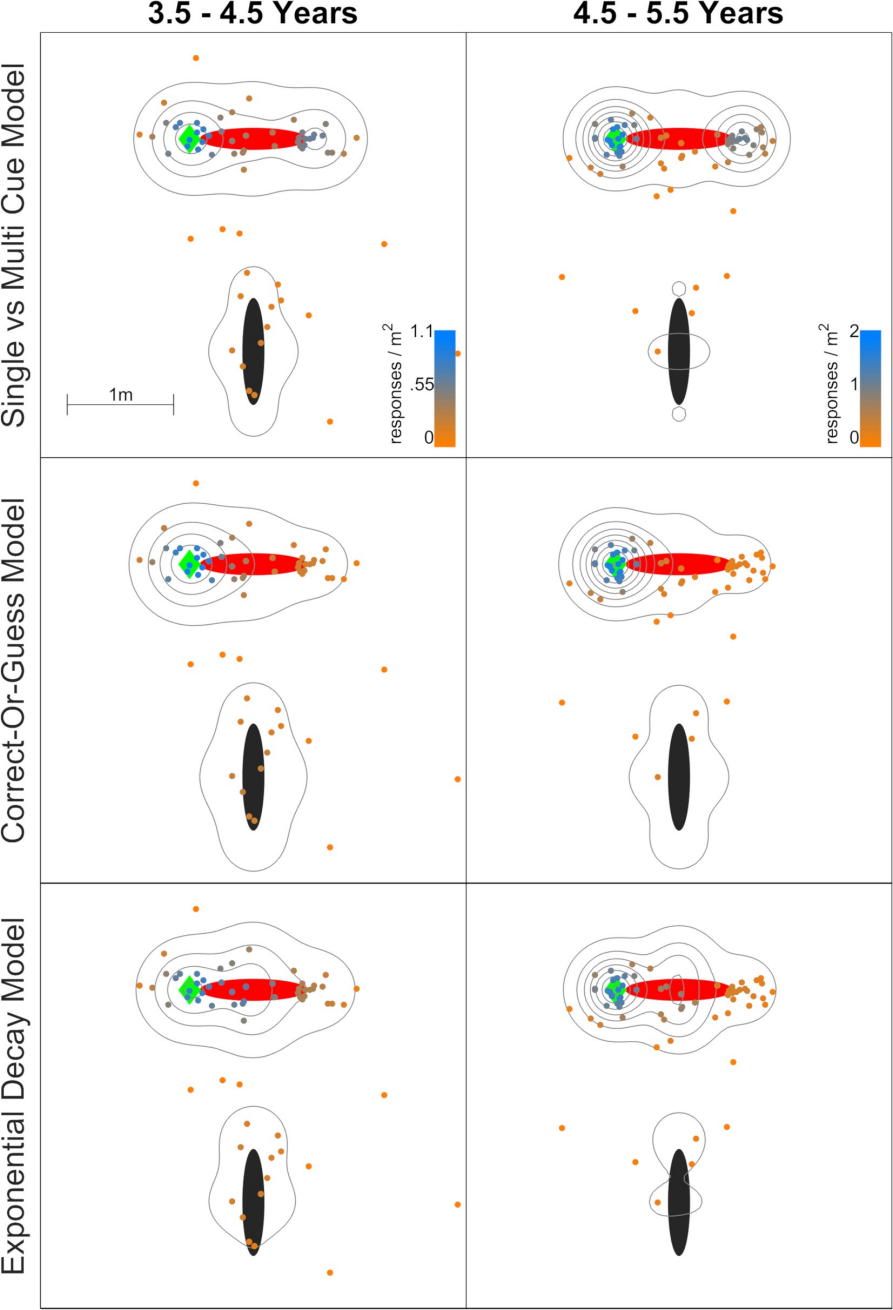
Model predictions and data for one target in the ‘Arctic’ dataset. The black and red ellipses are the landmarks. The green diamond (left of the red landmark) is the correct target. The contours show how these data are predicted to fall around the space based on the data with other targets, placed at probability density intervals of 0.25 per square meter, with the lowest at 0.1. Small colored circles are testing data, colored from blue (very likely) to gray to orange (very unlikely). The issue with the Correct-or-Guess Model and Exponential Decay Model can be seen on the right end of the red landmark: many data points fall there, but are assigned relatively low probability.

Fig 3 illustrates how the data and the cross-validation predictions from these three models (Fig 2) are distributed around the space in the ‘Arctic’ dataset. The Single- and Multi-Cue Model predicts good performance in terms of which landmark the target was near (p_1_) and whether it was on the side or end of that landmark (p_2_), but also a low rate of using the other landmark to prevent a local mirroring error (p_3_). In contrast, the other two models did not give especially high probability to the mirror targets (in this case, the rightward end of the red landmark). They do predict that errors will fall on the mirror end, but not in any special or particularly frequent way. This is the main reason why they do not cross-validate as well. To be more specific, for the 3.5–4.5 year olds, the Single- and Multi-Cue Model fits p_1_ = 0.79, p_2_ = 0.77, and p_3_ = 0.61. This means that the probability of a mirror error is 0.79*0.77*(1–0.61) = 24%. The Correct-Or-Guess Model fits p_c_ = 0.46. The probability of a mirror error is (1–0.46)/7 = 8%, since all errors share (1-p_c_) equally. The Exponential Decay model fits k = 0.058. The mirror error is 1.2m away, so the probability of a mirror error is proportional to e^-0.058*1.2^. After normalizing (so all targets sum to one), this equals 5%. The mirror errors in the testing data are too frequent for the Correct-Or-Guess or Exponential Decay model to capture effectively. S1 Text gives further illustration, explanation, and examples of how these three models function.

After visually inspecting Fig 3, we also wanted to be sure that it was not sensible to drop p_2_ and p_3_ entirely for the younger participants. Conceptually, this means that they remember which color landmark the target was near, but nothing further. A version of the Single- and Multi-Cue Model was run through the cross-validation process with p_1_ free, but fixing p_2_ = p_3_ = 50%. This cross-validated much worse, scoring 920.97 (3.5–4.5 years) and 670.42 (4.5–5.5 years) for the ‘Arctic’ data.

**Fig 4:**
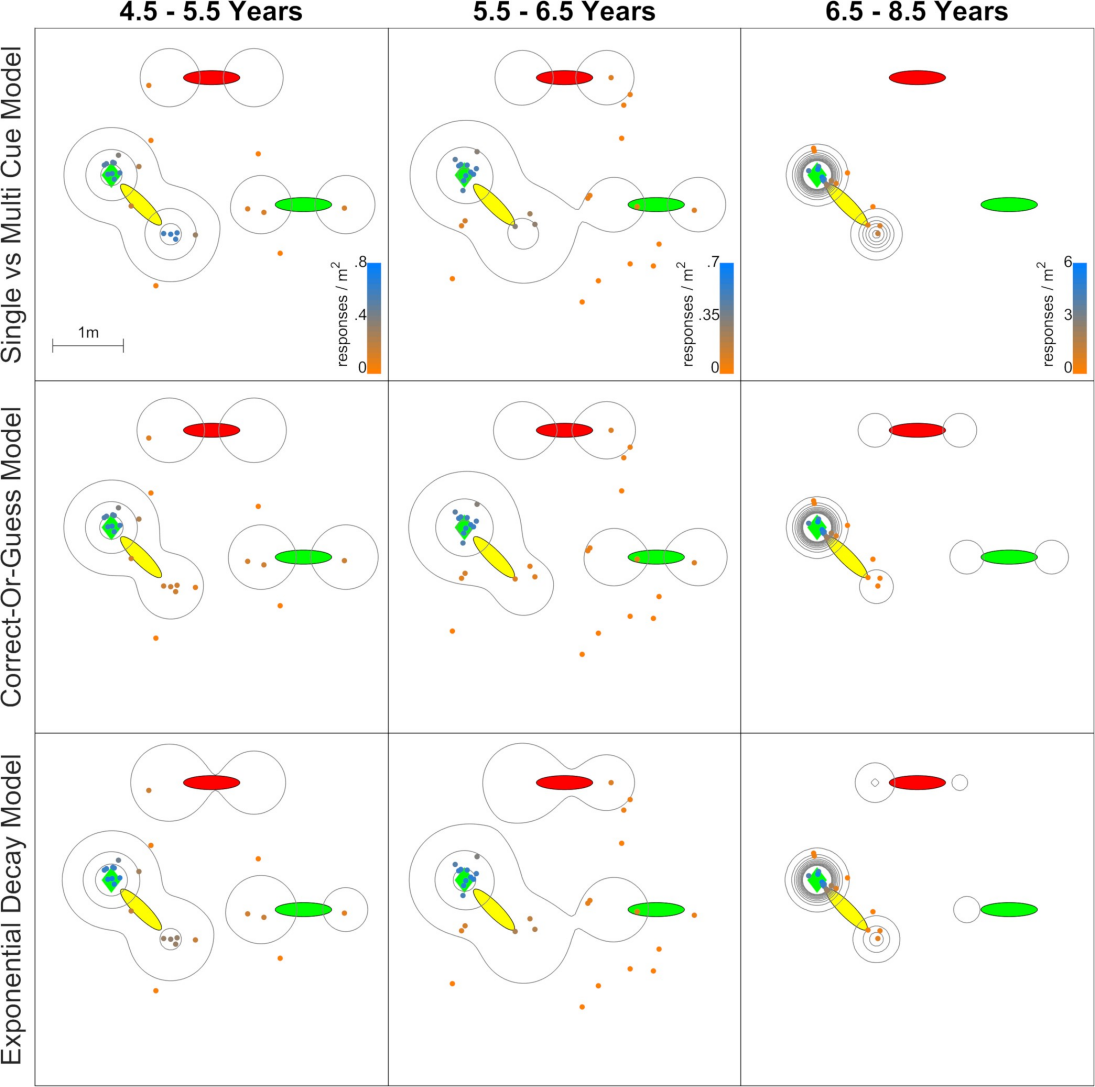
Model predictions and data for one target in the ‘Jetty’ dataset. The red, yellow, and green ellipses are the landmarks. As before, the green diamond (above and left of the yellow landmark) is the correct target. The contours show how these data are predicted to fall around the space based on the data with other targets, placed at probability density intervals of 0.25 per square meter, with the lowest at 0.1. Small colored circles are testing data, colored from blue (very likely) to gray to orange (very unlikely). The issue with the Correct-or-Guess Model and Exponential Decay Model is the same as before, failing to assign high probability to mirroring errors (down and right of the yellow landmark).

Fig 4 shows how the three main models apply to the ‘Jetty’ dataset. The ‘Jetty’ environment had several experimental differences from the ‘Arctic’, yet the results are very similar; the Single- and Multi-Cue Model is again preferred. In this experiment, there were no targets on the sides of the landmarks, only on the ends, so the p_2_ parameter was dropped. The data are again fit with a high p_1_ and low p_3_ for the younger ages. As above, this correctly predicts many mirroring errors and favours the Single- and Multi-Cue Model. S1 Fig plots all of the data, broken down by dataset and age range. The datasets are also given in Excel sheets in the S1 and S2 Data. Together with the ‘Arctic’ results, the ‘Jetty’ results suggest some basic generality to the Single and Multi-Cue Model, and therefore, this pattern is not unique to a single environment.

Since these three main models are structured differently, including different numbers of parameters, it is also important to verify that the models with less parameters can be selected by this procedure. For 100 runs, simulated data were generated from the Correct-Or-Guess Model with p_c_ = 1/3 and a standard deviation of 15cm. The targets and the number of trials (528) were matched to the 3.5–4.5 year olds in the ‘Arctic’. Each simulated dataset was submitted to the exact same procedure. The Correct-Or-Guess Model was chosen on 94% of the runs. The same was done with the Exponential Decay model, using *k* = 1. The Exponential Decay model was chosen on 98% of the runs. This verifies that the other models likely would have been selected by the model selection procedure if they were correct.

**Fig 5:**
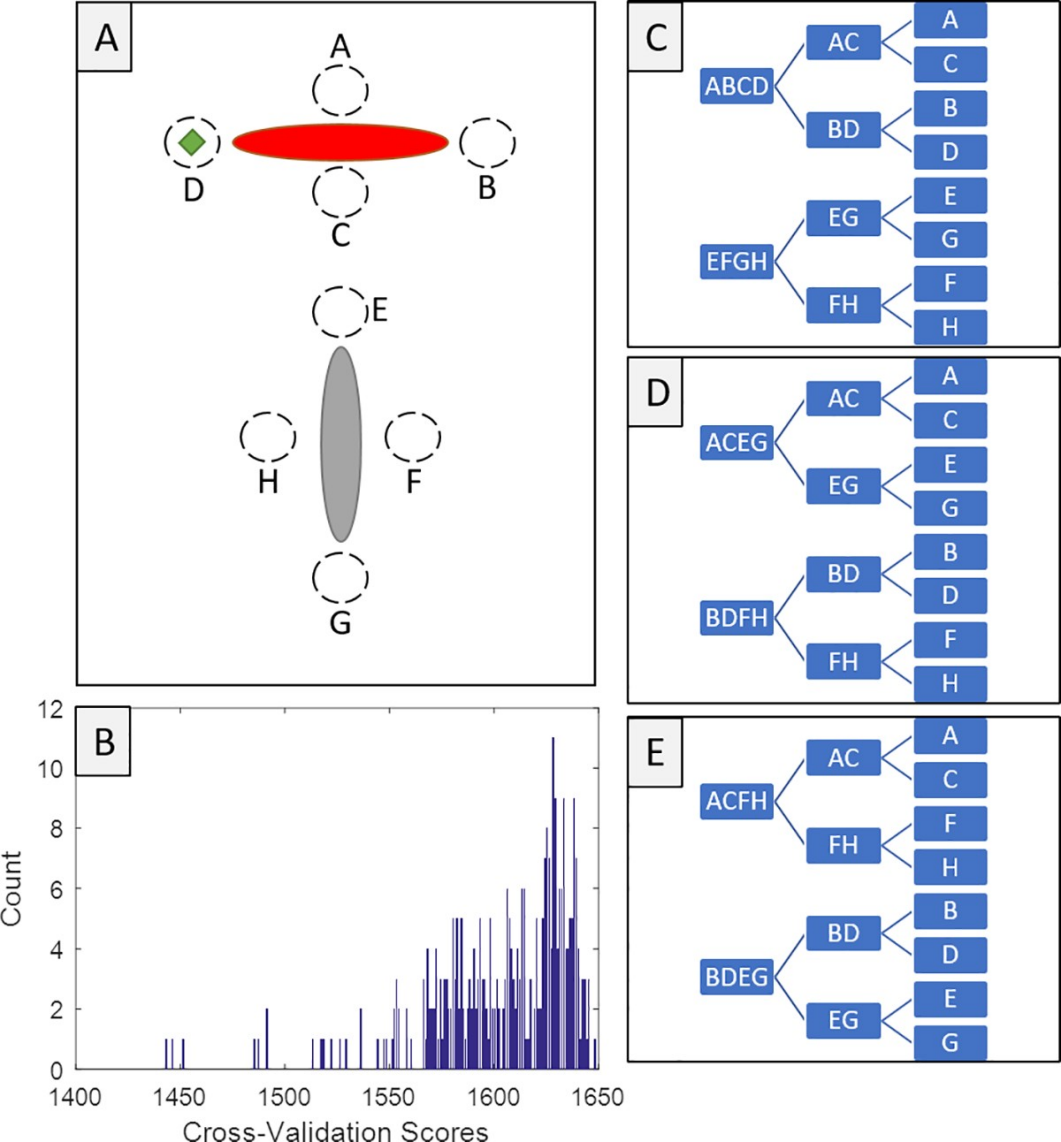
All possible ways of splitting the targets with three binary parameters were examined, favouring the main model presented here. (A) Display of the targets with letters for reference. (B) Histogram of the cross-validation scores from the 315 ways of splitting the targets. Only three models are within 100x the joint probability of the best-fitting. (C) The model with the best cross-validation score splits the targets by landmark, then side versus end, then local reflections (i.e. the Single- and Multi-Cue Model). (D) The second-best model splits targets in a way that reflections are still the third layer. The other two layers are similar but reversed in order, grouping by side/end then landmark. (E) The third best model also splits targets in a way that splits by side/end, then landmark, then reflections.

The Single- and Multi-Cue Model was also selected over the full family of structured-noise models for the ‘Arctic’ dataset. Because we are proposing a model with more parameters than would be expected from previous theory, it is important to make sure that these extra parameters are being used in the best way possible. We searched through all 315 possible models that use the same number and type of parameters (specifically three binary parameters) to split the eight targets into two groups of four, then two groups of two, and then two isolated targets. Fig 5B shows that a small sub-family of these models falls into the lower (better) range of cross-validation scores, leading to three top models. The best fitting one is the Single- and Multi-Cue Model. The other two are largely similar, at least in terms of our interpretation. For our purposes here, these top three models can be interpreted as having a separate p_3_ parameter for the relation to the other landmark. Further, all fit a relatively low p_3_ compared to p_1_ and p_2_. This means that, for example, when shown target D (in Fig 5A), all three top models predict target B to be the most likely error. Alternatives that placed less emphasis on local mirroring errors (e.g. using p_3_ to pair D with A, which could be resolved locally) did not cross-validate as well.

**Fig 6:**
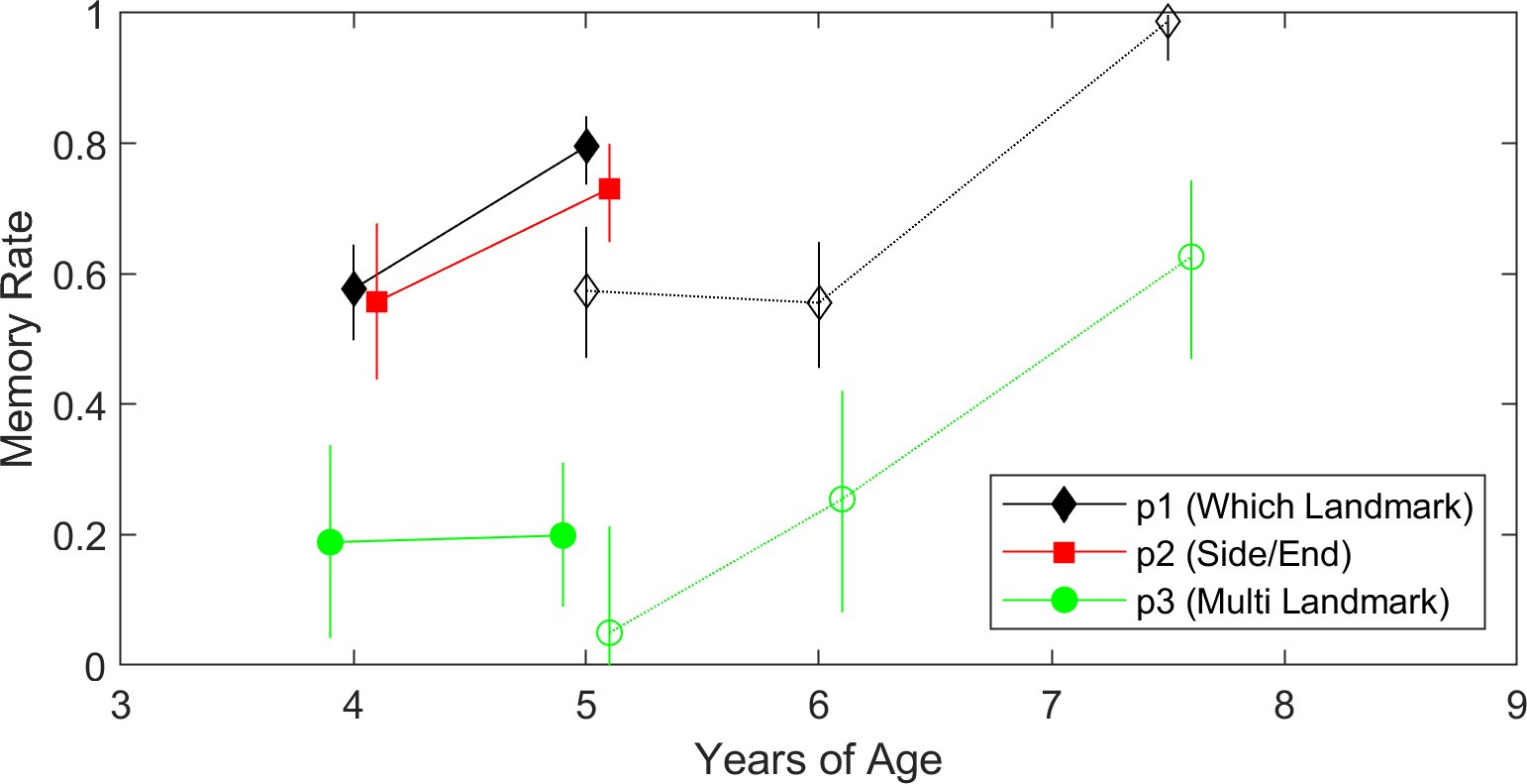
Model parameters by age group (displayed at the center of the range). Solid lines are from the ‘Arctic’ dataset and dashed lines from the ‘Jetty’. Error bars are 95% credible intervals. Since p_1_, p_2_, and p_3_ are all rates of correct response, and the two environments sometimes gave different chance levels, they were transformed into an inferred memory rate. For example, if a participant gives 60% correct responses with two options, we would work back to say that they were remembering correctly on 20% of trials and guessing on 80% of trials, leading to 40% correct by guessing and 20% correct by memory. A memory rate of zero would indicate chance guessing. The p_2_ line is only present for the ‘Arctic’ as the ‘Jetty’ only had targets on the ends of the landmarks.

Taken together, these analyses point towards a need for separate parameters to capture the rate of single- versus multi-cue encoding of target locations. It is not sufficient to model these errors as the more generic forms of noise in the comparison models; there must be special providence for mirroring errors that are predicted by just encoding against the nearest landmark. However, the interest in a particular model is often due to many factors, with the raw ability to fit and predict data being only one of them. A useful model should also have parameters that are easily interpreted and clearly relevant to a domain of study. To examine this further, the Single- and Multi-Cue Model was fit by Bayesian Markov Chain Monte Carlo methods [40]. The posterior parameter distributions were then used to answer the secondary questions:

What were the major sources of error in allocentric spatial memories at different ages? To answer this, Fig 6 shows the transformed parameter estimates at each of the age groups to generate a memory rate. It is evident that failing to use the far landmarks (p_3_) remained the most common error for all ages. In addition, the multi-cue memory rate only credibly rose above 50% in the oldest age group (6.5–8.5 years old). Note that this analysis would not be possible if only describing the data, for example, in terms of the average distance between target and response.

How can we now characterize the progression towards adult-like spatial memory? Further examining Fig 6, two additional features also stand out. First, if we consider each memory rate as a resource to be allocated, the way that young participants have done this is relatively sensible. If you could only remember two things about the target location, the landmark that it was nearest and the way it related to that landmark are reasonable choices to prevent very large errors. The estimates for p_1_ (which landmark) and p_2_ (side/end) were credibly higher than p_3_ (other landmark) for all age groups across both experiments. Second, while p_3_ was lower, the p_3_ credible interval was still entirely above-zero (i.e. they were not just guessing) for the youngest age group. Therefore, it is not that multi-cue recall only emerges in middle childhood, but that it becomes more frequent over a protracted span of childhood.

Were there any specific errors that actually became more likely as children got older? The posterior estimates from the Arctic dataset suggest that 3.5–4.5-year-olds and 4.5–5.5-year-olds had similar p_3_ rates, but that the older children had a higher p_1_ and p_2_. A mirror error happens with probability p_1_*p_2_*(1-p_3_). This indicates that mirroring errors become more common in the older children, but not at the expense of responses by the correct target; instead, at the expense of errors by the other landmark or incorrectly selecting the side versus end relation.

Was memory performance different in these two environments and does this point towards any possible new ideas to test further? The p_1_ memory rate estimate was credibly lower in the ‘Jetty’ environment, which is somewhat surprising. This environment had distal scenery that could be used for reorientation and a circular boundary that could be used to judge distance. The ‘Arctic’ did not have either. In general, little is known about what environments make spatial memory easier or harder in childhood. This may suggest that some spatial cues actually distract young children away from forming accurate memories rather than aid them. This raises interesting possibilities to test whether recent results arguing that young children reorienting in simple environments use multiple cues with Bayesian efficiency [41] would extend to more rich and complex environments.

**Fig 7:**
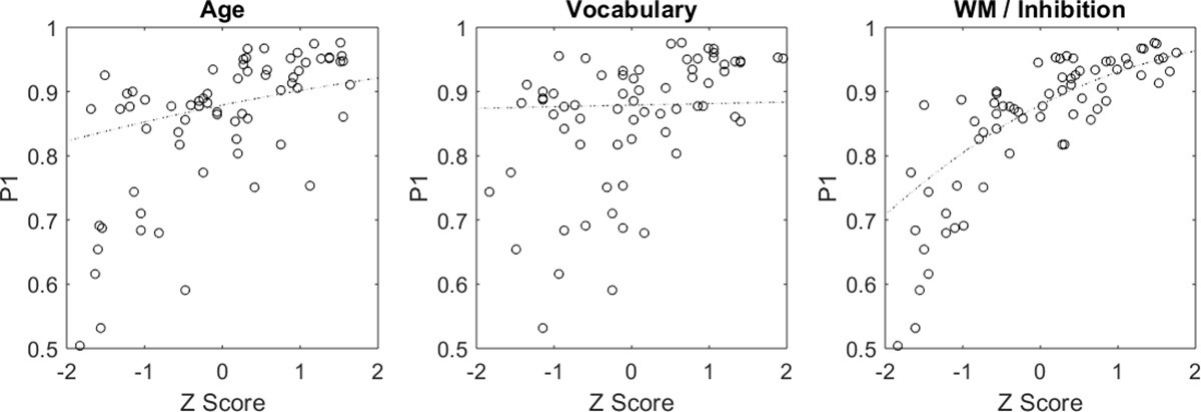
In a Bayesian regression, only one measure (Working Memory / Inhibition–Day/Night Task) shows a credibly non-zero effect on one model parameter (p_1_—which landmark). This could mean that the skills underlying the Day Night Task (working memory and inhibition) also help participants correctly choose which landmark the target was near, suppressing egocentric responses while maintaining allocentric representations in working memory. Circles are individual children.

Can we explain individual differences in spatial memory performance with more general cognitive measures–especially inhibition, which could play a role in suppressing less-accurate response strategies? To answer this, children in the ‘Arctic’ dataset were also given the Day-Night Task [42] and a basic Vocabulary measure [43]. The Day-Night task is a Stroop-like working memory and inhibition task that is appropriate for young children. They are asked to first say “day” when shown a sun and “night” when shown a moon, then later to say the opposite. In a Bayesian logistic regression on the p_1_-p_3_ parameters, Day-Night scores were a credibly non-zero predictor of p_1_ (which landmark; Fig 7, Table 2). In addition, the credible interval for this beta value still does not contain zero when switching from a 95% interval to a more conservative 99.5% interval: 0.033 to 0.60. Neither the vocabulary measure nor their chronological age were credibly non-zero predictors of any parameter (p_1_-p_3_) at the 95% level. This suggests that developing executive function could be a bottleneck in terms of developing spatial performance; they are entirely capable of the relevant computations, but perhaps face difficulty when organizing themselves to carry out the correct ones for the current task. Longitudinal datasets with more control measures would be immensely useful in answering this question (and many others) more definitively.

**Table 2:** Beta parameters relating the model’s key probabilities to predictor z-scores.

Parameter	Predictor		
	Age	Vocabulary	Working Memory / Inhibition
p_1_ (which landmark)	0.120 (-0.068 to 0.305)	0.016 (-0.157 to 0.196)	0.308 (0.122 to 0.500)*
p_2_ (side/end)	0.080 (-0.250 to 0.404)	0.159 (-0.161 to 0.511)	0.103 (-0.261 to 0.458)
p_3_ (other landmark)	-0.007 (-0.162 to 0.160)	0.035 (-0.127 to 0.194)	0.041 (-0.140 to 0.221)

*Credibly non-zero relation between predictor and model parameter (i.e. the 95% credible interval does not contain zero).

## Discussion

The model selection results point towards a need for a distinction between single- and multi-cue allocentric recall in order to capture and understand the different mechanisms used to remember a target location during childhood. Children at ages 3.5–6.5 frequently remembered which landmark a target was closest to and also how the target related locally to that nearest landmark (i.e. if it was on a side or an end of the oval landmarks). They also frequently made a specific error that can be predicted if they do not use the rest of the landmarks in the scene. The individual landmarks were ovals, which have two lines of symmetry. This required relating the target to additional landmarks in the overall scene in order to resolve which side/end of the landmark the target was on. For example, remembering the target as being “on the end of the green landmark” would ambiguously indicate two places since it had two identical ends. Participants frequently responded near that local mirror, the other end (or side) of the landmark. This is consistent with a failure to encode additional landmarks in their memory, which would prevent such errors. We also tried to model these errors without suggesting that they are special in any way (i.e. just a random guess that happened to fall on the mirror end, or a function of mirror ends being close to the correct target), but these attempts failed in cross-validation tests. Further, the tendency to only use one landmark fell systematically with age.

Integrating this new result with previous theory regarding egocentric reasoning [2–13], we arrive at a theory of spatial-cognitive development with three ‘levels’ in development: egocentric, single-cue allocentric, and multi-cue allocentric. This does not imply a stage-like progression, but three different modes in which children represent space that surrounds them with increasing sophistication. We theorize that children will tend to use earlier levels more often when they are younger and/or faced with a more difficult task (e.g. different instructions, different sets of landmarks, more or less time to respond). As the child matures and gains experience with each level, they become more likely to apply a more advanced level in a similar task. The first level, egocentric, involves encoding relations as a vector to the self. The second level, single-cue allocentric, involves encoding relations against a single salient landmark or scene feature. This was the predominant method for children under 6.5-years-old in the data presented here. The final level, multi-cue allocentric, involves encoding relations against multiple parts of the scene at once. This was the predominant method for children over 6.5-years-old. Effectively, this expands current theory from two levels to three, providing a more detailed trajectory of spatial-cognitive development. This could in turn provide a more useful framework for screening and intervention to promote spatial-cognitive development in early childhood [1]. This has its own merits, as the ability to flexibly use multiple landmarks in an allocentric representation is a very useful in everyday tasks. Spatial skills are also related to achievement in science, technology, engineering, and mathematics [44–48].

The view we take here contrasts with some specific parts of previous work [21, 28–30]. These papers take the view that allocentric spatial memories in early childhood are related to multiple landmarks, as previous studies have not shown any systematic reason to suggest otherwise. It is possible that results here stand in contrast because we used a variety of target locations, making it impossible for a response bias to imitate a successful multi-cue strategy.

This finding and general method (likely without VR) might also be useful to a much wider group of Biology researchers. The expansion paradigm has been employed across a large variety of species. To name a few: the common marmoset (*Callithrix jacchus jacchus*) [27], squirrel monkey (*Saimiri sciureus*) [49], orang-utan (*Pongo abelii*) [26], bonobo (*Pan paniscus*) [50], capuchin (*Cebus apella*) [50], and the mongolian gerbil (*Meriones unguiculatus*) [31]. Similar ideas have been employed to study the domestic dog (*Canis familiaris*) [51, 52], the rufous hummingbird (*Selasphorus rufus*) [53], and more. Many of these studies have suggested that these various organisms do not use more than the nearest single landmark. This could be further tested through an adapted version of the method here. Place two identical containers on the ends of an oval landmark. Place down another distinctive landmark. Always bait the container that is closer/further (counterbalanced across subjects) to the other distinctive landmark. The organism should learn to search the two containers near the oval landmark, but never learn to discriminate between them.

Comparing the parameter estimates from the two experiments suggests that the ‘Jetty’ was more difficult than the ‘Arctic’. This is unexpected because the ‘Jetty’ had more ways to encode locations. Based on this, we suggest that a recent developmental theory [41] needs further examination. This theory proposes that very young children routinely use multiple sources of spatial information in a rational Bayesian manner, gaining the full possible benefit from the presentation of multiple encoding methods. It seems difficult to reconcile this with the current results, but perhaps not impossible if the specific landmarks present are not equally informative across the two environments. If young children do reorient by combining cues with Bayesian efficiency, it would also be an exception to the general trend where they do not employ Bayesian cue combination in other settings [10, 54]. This tension should prompt further investigation. For example, young children may deal with one set of spatial cues with Bayesian efficiency but not another set of spatial cues.

Further, to be more specific, the ‘Jetty’ had both distal landmarks and a surrounding circular walkway. The ‘Arctic’ did not have either. Despite this, some level of multi-cue recall was seen in the ‘Arctic’ dataset, even in the youngest age group (3.5–4.5 years old). This suggests that the ability to use multi-cue recall is not entirely dependent on either distal landmarks [28] or the geometry of local boundaries [38]. This aspect of the results agrees with previous research on the use of multiple cues [21, 28–30].

A regression analysis further suggests that inhibitory control is a useful predictor of spatial performance. Specifically, it credibly predicted p_1_ (the parameter controlling the rate of egocentric or random responding). This could indicate that executive function forms a major bottleneck in terms of spatial memory development. Since executive function was not experimentally manipulated, we cannot be sure that this is a direct causal link. However, given the importance of executive function [55], this should be a point for future research to explore.

Further, as a more general point, the typical method of analysis for these kinds of data is to just report the rate of responses in the correct general area (plus any other general areas that are intentionally impossible to tell apart from the correct target) [9, 14, 29, 39, 41, 56]. This is an implicit endorsement of the Correct-Or-Guess Model. Results here suggest that these simple kinds of analysis can have significant limitations if there are multiple plausible strategies that a participant might use. In that case, it would be more informative to create a full model to fit to the data. There might be interesting patterns of errors that go beyond just being right or wrong.

The present study uses virtual reality to study spatial cognition. This can lead to biased estimation of egocentric distance if there is no opportunity to walk around the space [57]. However, participants here were frequently asked to walk through it. Beyond that caveat, despite a great deal of study, there is no specific reason to doubt the validity of virtual reality as a way to study spatial cognition; instead, there is a great deal of evidence that spatial cognition is the same in real and virtual environments [58–65]. This includes similar effects in young children [7, 8]. It also includes the transfer of training from virtual to real environments for neurological patients [66–68]. Despite that, it is unknown how tasks like ours here relate to other common spatial tasks, like mental rotation [69] or reorienting without a change in viewpoint [14]. Similarly, the relation between the skills on display here and spatial skills that Educators want to encourage needs more examination in the future.

An important avenue for future research is understanding which different cognitive and neural resources are deployed to enable the aspects of allocentric memory studied here. This may be investigated via relationships with other cognitive skills, and relationships with EEG or fMRI signals during the recall tasks. A powerful application of a model-based approach like the one used here would be relating individual parameter estimates (e.g. for p1, p2, p3) to individual behavioural and neural measures. Another important avenue for research is testing the potential of VR tasks of this kind for screening and interventions to improve childhood science and math education.

In conclusion, developing spatial cognition sometimes only represents the relation between a target location and one nearby single landmark. The resulting errors are not explained as well when modelling them as more generic errors, such as guesses that just happened to fall on the mirror reflection of the target. In that sense, capturing the single- versus multi-cue allocentric distinction is necessary to understand and predict spatial memory performance at different ages. Several aspects of allocentric spatial memory develop over childhood, including how often they remember which landmark the target was nearest–but a failure to use multiple landmarks remained the most common type of error. This leads us to theorize that spatial cognition has three developing ‘levels’: egocentric (self-based), single-cue allocentric (world-based but only using one nearby landmark), and multi-cue allocentric (world-based and using multiple landmarks).

## Methods

### Ethics statement

Ethics approved by the Ethics Committee in the Psychology Department at Durham University (14/05 –Development of navigation in virtual reality). The parents of participants gave written consent. Participants were asked to consent verbally.

### Participants

All participants were recruited around the Durham, UK area. In the ‘Jetty’ dataset, there were 12 children aged 4.5–5.5 years, 15 children aged 5.5–6.5 years, and 9 children aged 6.5–8.5 years. Not included here were two adults run to make sure the task was sensible (ages 28 and 25), both of whom had perfect scores on the categorical measures and a continuous error of <10 cm on average. The stopping rule for the ‘Jetty’ was to have at least 12 children in the lowest age bracket and 8 in the others, but to continue contacting families from all age ranges and gathering data from anyone who could be arranged during the data collection period. Since this procedure is relatively new, this stopping rule was chosen on the basis that 16 children is a relatively standard sample size (e.g. [70]) in studies of developing spatial cognition with far fewer trials (usually only 4 trials, compared to 14 here). In the ‘Arctic’ dataset, there were 17 children aged 3.5–4.0, 16 children aged 4.0–4.5, 17 children aged 4.5–5.0, and 18 children aged 5.0–5.5. The stopping rule was to have at least 16 children in each age group and to test any available siblings that wanted to participate even if their age bracket was filled. This was selected on the basis that 64 participants is high power (90%) for correlations of 0.4 and higher.

In the ‘Jetty’ dataset, the minimum age was chosen on the belief that children needed to be at least 4.5 years of age to reliably show allocentric recall at all [8]. Our choice to expand the age bracket for the oldest children (6.5–8.5 years) reflects a belief that the development of spatial cognition should start to slow around 7 years of age [2]. For the ‘Arctic’ dataset, the minimum age was chosen based on pilot data from a previous experiment that suggested the basic spatial task is too much of a motor demand (holding the wand still and pointing it accurately) for children under 3.5 years old [8]. To compare experiments, we wanted an overlapping age bracket, so we chose the range of 3.5–5.5. Because we wanted to look at correlation data with vocabulary and executive function, a larger sample size was desired.

### Apparatus

**Fig 8:**
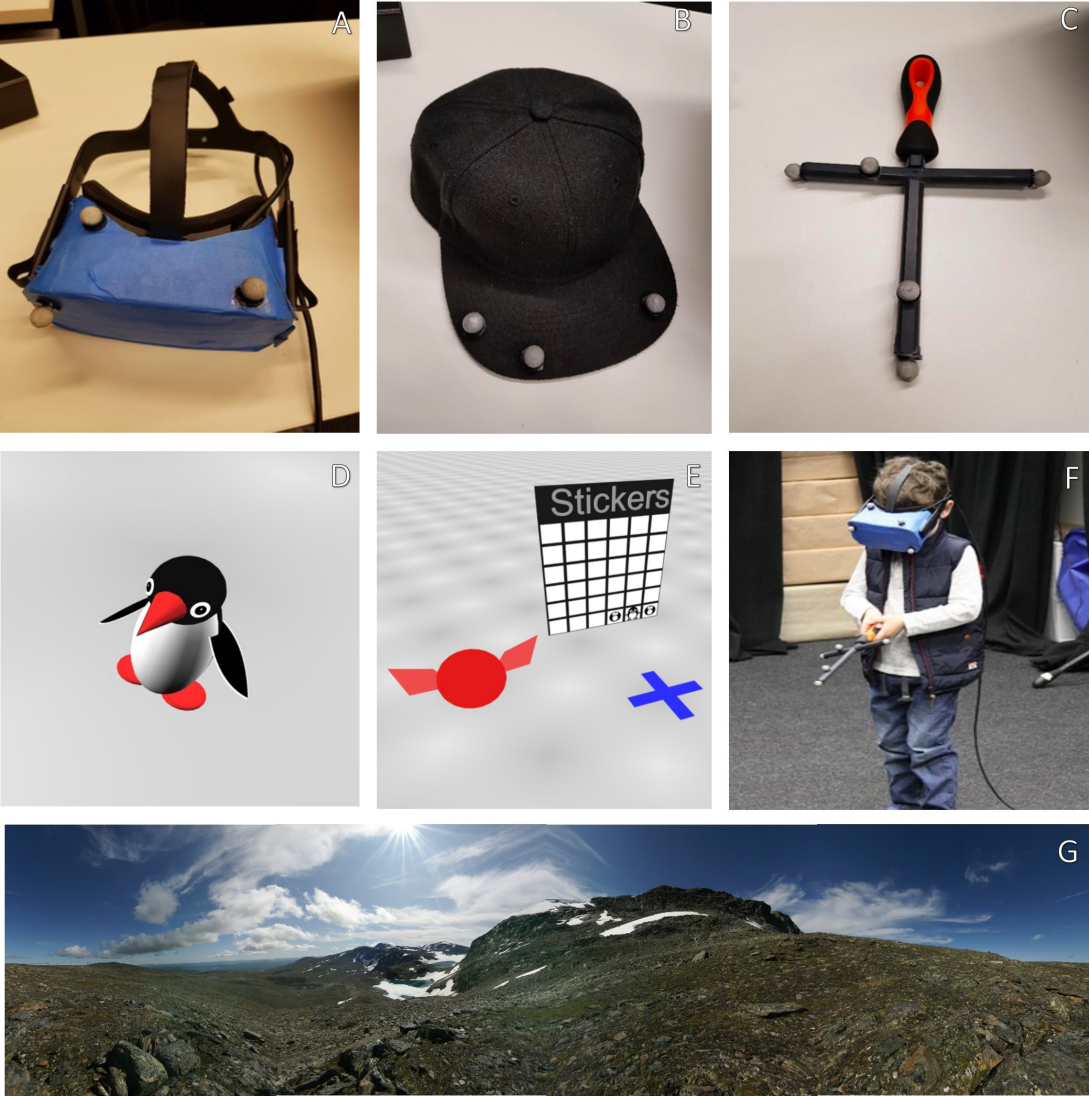
Photographs of the equipment and testing: (a) the headset, (b) the motion-tracked cap worn by the experimenter, (c) the ‘magic wand’, (d) the target object for the ‘arctic’, (e) the sticker chart used for the ‘Arctic’, plus the sprite representing the experimenter and the blue cross used to indicate where the participant should stand, (f) a child participating in the experiment with the equipment, and (g) the horizontal sections of the skybox used for the ‘Jetty’–an image that participants would see wrapped around the virtual world.

We conducted our experiments in a 5m x 9m laboratory equipped with 16 infrared Vicon Bonita cameras. The cameras could motion-track by tracking reflective markers and had a capture rate of 240 frames per second at <1mm resolution. The markers were attached to a head-mounted display (Oculus Rift; see Fig 8A), a wand, and a cap. The experimenter wore a cap (see Fig 8B), and this cap appeared in VR as a sprite–a large circular figure with ear-like structures on it. The sprite was used because young children in the piloting phase of a previous project [8] found it upsetting to hear the experimenter’s voice (explaining the game and giving instrucitons) with no visible source. The participant held a pointer (see Fig 8C) constructed out of a screwdriver handle and PVC cylinders. The Rift has a field of view of 110 degrees and a resolution of 2160x1200 with a refresh rate of 90Hz. The headset can be adjusted according to the head size of the participant to ensure that it fit properly.

### Virtual environments

The virtual environments were programmed in Vizard (WorldViz, Santa Barbara, CA) (Fig 1). Both involve a large circular arena where targets appeared, with a donut-shaped area around it where participants stood to encode and recall the targets. Both also involved elongated spheres as landmarks in the target area. The landmarks were different colors. They were set apart by a minimum of 1.5m in an effort to make it clear that they were distinct landmarks and not parts of a larger structure. In both experiments, there was also a large flat X that lay on the ground surface and could be moved to different places by the program (Fig 1E). This was used whenever we asked participants to stand somewhere (“Can you come stand on this cross for me?”) or when telling them that they were going to be ‘teleported’ somewhere. To make the 3D nature of the space easier to see, both environments had a light checkerboard pattern on the surface of the ground plane (Figs 1 and 8E).

In the ‘Jetty’ dataset, there were three landmarks set at oblique angles. Participants were screened for colorblindness by asking if they could tell which was green, which was red, and which was yellow. In the only case where the participant was not able to do this, the landmarks were changed to white, gray, and black. There was an orienting ‘skybox’, meaning an image that is wrapped around the virtual environment–something that the simulation shows if there are no other virtual objects in the way. It was a mountain scene adapted from a photograph taken in Iceland (Fig 8G). This cue is rendered at infinite distance, so it can be used to orient but not to measure distances. The encoding and retrieval area was displayed as a jetty that the participants stood on, raised 50cm above the water. This was also a spatial cue, but opposite in use–it could be used to measure distances to its edges, but it was not possible to orient to it. The target was a small duck. To help participants differentiate trials and maintain interest, the duck appeared in different, random primary colors on each trial. It also spun slowly in place to attract attention.

In the ‘Arctic’ dataset, there were two landmarks set at a right angle. The landmarks were longer (1m) than those used in the first experiment (0.8m) in order to make the two ends more distinctive and further apart, in case motor noise was a severe problem for the younger children. The encoding and retrieval area was not marked in any visible way. The skybox was a uniform light blue, which could not be used to orient. The target was a small penguin. We were concerned that the younger children may not know the word ‘penguin’, so the target was named Steve and this was used for all of the instructions given to participants. To help participants differentiate trials and maintain interest, Steve’s torso changed to random primary colors on each trial. To attract attention, Steve also moved his head and wings up and down a small amount.

### Procedure

#### Training and warm-up trials

In the ‘Jetty’ dataset, only a very brief pre-training procedure was needed before entering the VR. Participants were simply asked to stand up straight and hold the wand with both hands. In the ‘Arctic’ dataset more extensive pre-training was needed for testing a younger age range. Children were asked to point, using their wand, for three seconds at a real stuffed animal/penguin while not wearing the headset. For the first four of these practice trials, the stuffed animal was openly visible on the floor. For the next four, it was hidden under a towel to imitate the penguin hiding underground in the actual experiment. If needed, the experimenter would give feedback and would model the requested posture for the participant to mimic.

After this practice, for both experiments, children wore the headset and were immersed within VR. During the first 4 trials in VR, participants practiced pointing in the new environment. For these trials, the target simply went to a new place and the child was asked to point at it and hold the wand still until a response registered. When the wand (Fig 8C) was pointed at the ground plane, a white circle appeared where the wand was pointed. As with all parts of the experiments, a response registered when the indicated location was stable within a 20cm area for 2 seconds. During movement beyond this 20cm range, the white circle would expand. When the wand was being held still within the 20cm range, the circle would shrink. This was done so that the circle would collapse entirely just as the response registered. Trials continued until 4 responses were registered within 50cm of the target.

The next trials were designed to help the participants practice recalling locations from memory. The target moved to a new location and then disappeared. (In the ‘Jetty’, it simply faded from view. In the ‘Arctic’, this was animated as Steve digging underground.) After a 3 second delay, participants were instructed to point to the target location. The character (duck/penguin) then re-appeared but a marker was left where the response registered.

As with all trials, this was narrated by the researcher. First a generic affirmation was given: “Good job!”. Next, a positive aspect of the response was noted: “You held the wand really still”, “You pointed by the [green] one, and look, it was over by the [green] one”, or “You pointed by that end of the [green] one, and look, it was actually on that end of the [green] one.” Then, if the response was not on the correct end of the correct landmark, any constructive feedback was given as future goals: “You pointed by the [green] one, but he was actually over by the [yellow] one. For the next one, I bet you can remember which one he is by”, or “But look! He was on the other end! For the next one, let’s see if we can also remember which end he is on.” This again went on until 4 responses were registered within 50cm of the target. Later, for targets far away, feedback was more generic: “Good job! Look where you pointed” followed by “He was right there” for responses within a small error versus “and look where he was” for larger errors. General encouragement was also given as needed, such as “You’re doing great.”

Next, they were presented with another 4 trials designed to show them how the teleporting worked. The target moved to a new location and a green cross appeared. The experimenter explained that the target would hide and then we (both the participant and experimenter) would get ‘teleported’, meaning that the computer would move us over to the green cross. The child was asked to imagine what the scene would look like from the green cross and to take a careful look at the location of the target. The target hid. The screen faded slowly to black over a period of 1.25 seconds. The participant’s viewpoint and the experimenter’s avatar in the VR simulation changed (i.e. the experimenter ‘teleported’ with the child). The screen slowly faded back up over another 1.25 seconds. The child was asked to look where they were now and then to point to the target. There was no criterion for advancement. The size of the teleports around the donut-shaped encoding/response area increased over these trials: 22.5, 45, 135, and 180 degrees from their original position.

Interspersed throughout this procedure and the data collection trials were requests to move by walking. The experience of walking in VR can help participants understand the scale of the space correctly [58]. For these parts, a blue cross appeared on the ground and participants walked over to it with the experimenter. This took place after every third trial. In the ‘Arctic’ experiment, to help motivate the younger children, a virtual sticker chart appeared showing how many trials they had done. Also, there were scheduled breaks. 3.5–4.5 year old children were given mandatory breaks after every 6^th^ trial to receive actual stickers and place them on a printed picture of a penguin. 4.5–5.5 year old children were asked if they wanted to take a break and put real stickers on their chart or if they wanted to keep playing and put the stickers on later. In the ‘Jetty’, with older children, breaks were given in a less structured way. Any breaks requested by the participant were given immediately and the experimenter would occasionally offer them if the child seemed distracted or in need of one. There was no specific activity or reward during these breaks.

#### Data collection trials

In both experiments, participants were asked to look carefully at the location of the target. When the participant was ready, the target disappeared. In the same procedure as above, they were then teleported to a different viewpoint from which they had to point to where the target was. The main difference is that they were not shown where they would be teleported to (no cross on the ground). After pointing, the target re-appeared and their choices were narrated as before.

In the ‘Jetty’, teleports consisted of rotating 45, 60, 75, 110, 140, or 170 degrees, on a total of 30 trials. There were 14 trials where the target locations were near the landmarks (blue “x” in Fig 1C), four of which were on the two ends of the red landmark. Twelve additional trials tested targets classified as “far targets” in the upper and lower halves of the arena, between landmarks (green “x” on Fig 1C). The remaining four trials tested the specific location that was the farthest from the landmarks and the walkway (lower third, middle horizontally on Fig 1C). Each teleportation magnitude was used evenly (i.e. 5 times), with the constraint that the rotations used for any given target category above were evenly split as rotations under 90 degrees and over 90 degrees.

In the ‘Arctic’, teleports consisted of rotating 30, 40, 50, 60, 105, 125, 145, or 165 degrees. There were a total of 16 trials near the landmarks, with each target repeated twice. The other 8 targets were each used once. This made for a total of 24 trials. Each teleport amount was used evenly (3 times). All 8 rotations were randomly paired with the 8 far trials. Each near target had one rotation under 90 degrees and one over 90 degrees.

### Additional measures

#### Location description task (Jetty)

After the very last data collection trial, the participant was unexpectedly asked to turn away from the duck and to "tell [the experimenter] with words instead of pointing" where the duck was. Their response was noted and then they were asked to turn back around, facing the landmarks and the target. The experimenter said that they were keeping their eyes closed (to further discourage pointing) and asked the participant if there was anything else they could say about where the duck was. Feedback was not given on this last trial.

#### The British picture vocabulary scale III (BPVS) (Arctic) [43]

The BPVS tests receptive vocabulary for Standard English in children between 2 years and 6 months old to 6 years and 11 months old. It can indicate language development and vocabulary knowledge and takes 5–8 minutes to complete. The procedure largely consists of asking the child to identify which of four displayed pictures corresponds to a given word. It was administered according to the standard procedures and instructions in the manual.

#### The Day-Night Task [42] (Arctic)

The Day Night Task is a measure of inhibitory control (IC), which tests if an individual can suppress an acquired /dominant response and replace it with a competing response (Montgomery & Koeltzow, 2010). The Day-Night task is a simplified version of the Stroop Test and is often used with young children. It involves a set of cards that either have a picture of the sun or the moon. In the first part of this task (forward condition), the children were instructed to say “day” when they saw a sun card, and “night” when they saw a moon card. In the second part of the task (reverse condition), which was taxing for both memory and inhibition, children were asked to say “day” when they saw the moon, and “night” when they saw the day. Children were timed and the number of mistakes was recorded.

#### The behavioral rating inventory of executive function (BRIEF)—preschool version [71] (Arctic)

The BRIEF-P is used for children between 2 years, 0 months to 5 years, 11 months. In this study, the Parent Form questionnaire of the BRIEF was used. This form asks questions about how often their child’s behaviors were problematic over the past six months. It has good internal consistency (0.80–0.95) and moderate test-retest reliability (0.78–0.90). These data are reported in the SI, for the sake of completeness, but they were not interpreted here.

### Models

The proposed Single- and Multi-Cue Model has four implicit stages. First, the children need to remember which landmark the target was closest to. With probability (1-p_1_), they fail and select a random Gaussian near the other landmark. With probability p_1_, they remember correctly and move to stage two. Stage two involves remembering whether the target was on the side or end of the landmark. With probability (1-p_2_), they fail and select a random Gaussian among the non-side or non-end targets that are near the correct landmark. With probability p_2_, they remember correctly and move to stage three. At stage three, they need to use the relation to the other landmark to resolve the local symmetry. With probability (1-p_3_), they fail and select the Gaussian over the local mirror of the target. With probability p_3_, they remember correctly and select the Gaussian over the correct target location. At this point, they have selected a general area represented by the selected Gaussian. However, they are likely to have some additional small sources of noise, such as motor error. Their final response is modelled as a draw from the selected Gaussian. All of the Gaussians share a single variance that applies along both axes with no correlation (i.e. is circular).

The Correct-Or-Guess Model has two implicit stages. First, the children need to remember the correct location. With probability (1-p_c_), this fails and they select a random incorrect Gaussian. With probability p_c_, they select the correct Gaussian. In the final stage, their response is modelled as a draw from the selected Gaussian.

The Exponential Decay Model also has two implicit stages. The pre-normalized probability of selecting each Gaussian is e^-kd^, where *d* is the distance between the target and each response area’s center, in meters, and *k* is a free decay parameter. These probabilities are then all divided by their sum so that they sum to 100%. A Gaussian is drawn based on these normalized probabilities, then the response is modelled as a draw from this Gaussian.

The 315 structured-noise models are generalizations of the proposed model. In the first stage, they are faced with two sets of four Gaussians. One set contains the correct target. They need to select this set. With probability (1-p_1_), they fail and select a random Gaussian from the incorrect set. With probability p_1_, they remember correctly and move on to stage two. In stage two, the selection is narrowed from four to two. In stage three, it is narrowed from two to one. These structured-noise models search through all 315 possible ways of grouping the Gaussians into a hierarchy that allows this.

Each of the models was fit in the cross-validation procedure with the fminsearch function in Matlab, minimizing the negative sum of the log probability of the training data. The testing data were then sent through the same function with the fitted parameters to calculate their associated score.

### MCMC analysis

For Fig 6, the Single- and Multi-Cue Model was submitted to slice sampling with no explicit priors. There were 10,000 samples drawn for each age group, seeded with a maximum likelihood estimate.

For an overall model relating the p_1_-p_3_ parameters to the three predictors, a Bayesian logistic regression was used (see Fig 7). This means that each parameter (which is a probability and therefore between 0 and 1) was the inverse cumulative normal distribution of a real-valued parameter. That real-valued parameter was then the sum of five figures: mu + b_1_*Z_1_ + b_2_*Z_2_ + b_3_*Z_3_ + E. Z_1_ was the z-score of their chronological age. Z_2_ was the z-score of their vocabulary score (the total number of correctly-answered questions in the BPVS). Z_3_ was a combined score for the Day-Night task, the z-score of the rank of their time plus the z-score of the rank of their errors in the second round with reversed instructions. (Ranks were used due to large outliers.) E was a normally distributed error amount with a mean of zero and a precision of tau. This structure was repeated three times, once for each of the p_1_-p_3_ parameters. Only one explicit prior was used: tau was given an exponential prior with a mean of 100. The other parameters implicitly have a flat (completely non-informative) prior. Four chains of 5,000 samples were drawn, each with 1000 discarded as burn-in.

For the logistic regression, we chose to report credible intervals rather than a Bayes factor. This was done because it is, in our opinion, more appropriate for the present stage of understanding of these data. A Bayes factor is used to compare two (or more) specific models with specific prior distributions to see which one has a better average fit to the observed data. Best practice for a Bayes factor involves creating a relatively small number of *a priori* restricted models to compare. In other words, the best-practice use of a Bayes factor involves both the specification of prior distributions and the prior selection of restricted models to be compared. We did not believe this to be appropriate in this situation. In contrast, we report credible intervals that were calculated without any explicit prior distributions and without the prior selection of restricted models. This results in unbiased interval estimates of an unrestricted model. Future work can use the results for a more principled Bayes factor comparison.

## Supporting information

S1 Data Arctic dataset. (XLSX)

S2 Data Jetty dataset. (XLS)

S1 Fig All Data in figure form. (DOCX)

S1 Text Additional model explanation. (DOCX)
