# Supplementary material for: Coding Locations Relative to One or Many Landmarks in Childhood
Source: PLoS Comput Biol. 2019 Oct 28;15(10):e1007380. doi: 10.1371/journal.pcbi.1007380 (PMC6816551; doi:10.1371/journal.pcbi.1007380)
Supplement: S1 Fig — (DOCX) [file pcbi.1007380.s003.docx]

**SI Figures**

These figures plot all of the responses in the two datasets, ‘Arctic’ first, separated by age range and then panelled by target. The green diamonds are targets and gray dots are responses. The ovals are landmarks. It is perhaps easiest to informally see the basic effect that this paper discusses in these displays. In Figure B, the distribution of responses is very sensitive to the nearest landmark (top half versus bottom half of panels), and also to whether the target was on the end or side of a landmark (left half versus right half of panels). However, the targets that were reflections of each other (touching panels) across the local landmark lead to very similar response distributions. This is expected if participants are largely remembering target locations by only using the nearest single landmark. The main text confirms formally that this is a sensible interpretation.

**Figure A**

**Figure B**

**Figure C**

**Figure D**

**Figure E**
