# Supplementary material for: Coding Locations Relative to One or Many Landmarks in Childhood
Source: PLoS Comput Biol. 2019 Oct 28;15(10):e1007380. doi: 10.1371/journal.pcbi.1007380 (PMC6816551; doi:10.1371/journal.pcbi.1007380)
Supplement: S1 Text — (DOCX) [file pcbi.1007380.s004.docx]

**S4 Text – Additional Model Explanation**

This appendix exists to help the reader understand the three main models (Single- and Multi-Cue, Correct-Or-Guess, and Exponential Decay) in more detail.

All three are Gaussian mixture models. This means that for *n* Gaussian distributions indexed by *i*, the probability density at a single point *X* is

(A1) $\sum_{i=1}^{n} W_{i}\varphi_{i}\left( X \right)$

where *W_i_* is the weight given to each Gaussian and $\varphi_{i}\left( X \right)$ is the *i*th Gaussian’s probability density function. Further, *W_i_* follows the constraints 0<*W_i_*<1 and $\sum W_{i}=1$. For example, Figure A1 is a 3D plot of two Gaussians in a mixture (just to illustrate what a Gaussian mixture model is like). The first is weighted at 75% and the second at 25% (*W*_1_ = .75, *W*_2_ = .25). The first has a mean at (2,1) and the second has a mean at (-2,0). Both have a variance of one in each direction and a covariance of zero. This means that there are local modes at (2,1) and (-2,0), but the probability at (2,1) is higher. In addition, the probability density at (0,0) can be found by finding the probability from the first and second Gaussian, then taking a weighted average. The probability density at (0,0) from the first is .013 and from the second is .022. The probability density under the mixture is

0.75 * 0.013 + 0.25 * 0.022 = 0.015

In broader applications, Gaussian mixture models can be fit by changing the weights, means, variances, and covariances for the Gaussians in the mixture. Here, we are effectively limiting ourselves to mainly working with the weights and variances.

Figure A1. Example of a Gaussian mixture distribution.

For all of these models, several things were constrained:

- The means of the different Gaussians were all the correct targets (eight for Arctic, six for Jetty). This is because the responses tended to cluster around the landmarks, so these means can capture the vast majority of the data.
- The variance along the x axis was the same as the variance on the y axis (i.e. in both directions on the ground plane). This was done mainly because it would have been difficult to interpret on its own and would increase the number of parameters significantly. This is also the reasoning behind the next two constraints.
- The covariance was zero. Together with above, this means all the Gaussians are symmetric ‘bell’ or ‘circle’ shapes.
- The variance for all the different Gaussians was equal.
- To deal with outliers, all of these mixtures reserved 99% of the probability for the Gaussians and 1% for a uniform distribution. This is also done through weighted averaging, like formula A1 but substituting 1/9π (where 9π is the area of the arena) for $\varphi_{i}\left( X \right)$.

In a more compact equation form:

(A2) $P\left( Rx,Ry | T,\boldsymbol{W},\sigma\right)=\frac{.01}{9\pi}+.99 \sum_{i=1}^{8} \left[ W_{i}\varphi\left( \left[ Rx,Ry \right] | M=\left[ {Tx}_{i},{Ty}_{i} \right], \Sigma=\left[ \begin{matrix} \sigma^{2} & 0 \\ 0 & \sigma^{2} \end{matrix} \right] \right) \right]$

Where *Rx* is the x-axis position of the response, *Ry* is the y-axis position of the response, *T* is the target index, ***W*** is the vector of weights, *σ* is a free variance parameter, $\varphi_{i}$() is the bivariate normal probability density function, *Tx_i_* is the x-axis position of target *i*, and *Ty_i_* is the y-axis position of target *i*. The sum is up to 8 for the Arctic (shown here), because there were 8 targets, but up to 6 for the Jetty, because there were 6 targets.

The vector of weights is determined differently for the three models. The Correct-or-Guess model assigns the Gaussian over the correct target a weight of p_c_ and gives all other Gaussians the weight (1-p_c_)/7 for the Arctic or (1-p_c_)/5 for the Jetty. The exponential decay model assigns weights proportional to e^-kd^, where *d* is the distance to the correct target in meters, *k* is a parameter that controls how concentrated the responses will be on the correct Gaussian, and *k*>0. For example, suppose *k* = 2 and we want to know the weight *W_i_* of a target that is 3m away from the correct target. In that case, $W_{i}\propto e^{-6}$. To enable the extra flexibility to specifically predict mirroring errors, the Single- and Multi-Cue model has more parameters. The parameter p_1_ is the probability of selecting the correct landmark, p_2_ is the probability of selecting the side versus end correctly, and p_3_ is the probability of correctly selecting between the target’s side/end instead of its mirror across the landmark (i.e. multi-cue recall). For example, suppose the correct target is by the red landmark, on an end, and specifically by the west end. We want to find the weight *W_i_* of a target that is by the red landmark (correct), on an end (correct), but on the east end (incorrect). This means that $W_{i}=p_{1}p_{2}\left( 1-p_{3} \right)$. We will give some example distributions below.

What is the point of the alternative models? We are looking for evidence that children at this age sometimes employ a single-cue recall strategy. At one extreme, where all they do is single-cue recall, we would expect half the responses on the target and half at the local mirror. At the other extreme, where all they do is correctly use multi-cue recall, there would be no responses at the local mirror. However, there can be intermediate situations (and indeed there were). In that case, this idea predicts that errors will be somewhat concentrated at the local mirror of the correct target. That statement needs to be quantified and compared to something so that it can be verified. In other words, when looking at the data, it is obvious that some errors fell on the local mirror. But do those responses count as a ‘concentration’? Just by introducing noise, we could occasionally see responses at the local mirror. To answer, we compare against alternative models that predict some local mirroring errors as well, but as a function of generic noise rather than a single-cue strategy. This generic noise is either based on pure guessing (Correct-or-Guess) or based on distance to the target (Exponential decay). This allows us to be sure that we are not simply taking any local mirroring error as indicative of a single-cue strategy, but instead that such errors are concentrated enough to warrant separate modelling.

We next want to give some examples of what kind of response distributions each model can give as a function of different parameter settings (Figures A3-A5). First, we need to explain how these charts can be interpreted (Figure A2). Dark blue is zero probability. Bright yellow is a probability density of four per meter squared. The correct target is placed just below the center.


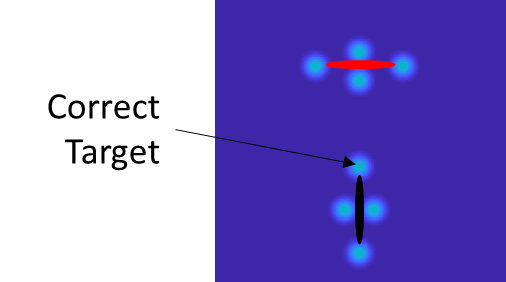


Figure A2. Interpretation guide for Figures A3-A5.

To help see the actual predictions clearly, the target and landmarks are omitted from Figures A3-A5. However, the frame used to draw the distribution is the same as Figure A2.

Figure A3 gives some examples of how the Correct-Or-Guess model can vary with different parameters. Rightwards, the probability of choosing the correct Gaussian (p_c_) increases. Downwards, the variance of the Gaussians increases. This results in a variety of different possible response distributions. For example, the bottom left essentially just places more probability nearer to the landmarks without much further distinction. The bottom right is very near to a large Gaussian centered on the correct target. Conceptually, these are the kind of patterns that can be predicted while obeying the constraint that every incorrect Gaussian is equally likely (i.e. you either know or guess randomly).

Figure A3. Example distributions predicted by Correct-Or-Guess.

Figure A4 gives some examples of how the exponential decay model varies. Rightwards, the decay rate *k* increases. Downwards, the variance of the Gaussians increases. This again results in a variety of different noise distributions. For example, in the top right, the correct target is frequently confused with the two nearest targets. Conceptually, these are the kind of patterns that can be predicted while obeying the constraint that closer incorrect Gaussians must be more likely than further ones.

Figure A4. Example distributions predicted by the Exponential Decay Model.

Together, the Exponential Decay model and the Correct-or-Guess model represent a variety of different ways that generic noise can be distributed. What they lack is a way to specifically predict a high rate of mirroring errors. This is where the Single- and Multi-Cue model becomes relevant. Figure A5 illustrates what happens if we fix the variance at .01. Rightwards, p_1_ and p_2_ increase. Downwards, p_3_ increases. In the top left is random guessing among the different Gaussians. In the top right is a pure single-cue strategy. In the bottom right is a pure multi-cue strategy. In the middle are intermediate levels of all three. Towards that middle region is where all of the best fits were found.

Figure A5. Example distributions predicted by the Single- and Multi-Cue Model.

As a whole, these are not an exhaustive search of all possible models that could be applied to these data (such a task is impossible). However, they are all relatively similar in their core mathematical mechanics and the conceptual difference between them is relevant to our theory. For example, no model gets the advantage of moving the means to better accommodate the data; the introduction of the p_3_ parameter is motivated by capturing single-cue recall strategies. It (always) remains possible that a better model will be found in the future, but for now, we have shown that two generic versions of noise cannot explain these data as well as a single-cue strategy.
